# Synchronous Solid Pseudopapillary Tumor and Insulinoma in an Adolescent MEN1 Patient Presenting with Diagnostic Dilemmas

**DOI:** 10.4274/jcrpe.4799

**Published:** 2017-12-15

**Authors:** Ahmet Uçar, Banu Özgüven, Muharrem Battal, Ferda Alparslan Pınarlı, Evrim Özmen, Aylin Yetim, Yasin Yılmaz

**Affiliations:** 1 University of Health Sciences, Şişli Hamidiye Etfal Training and Research Hospital, Department of Pediatric Endocrinology and Diabetes, İstanbul, Turkey; 2 University of Health Sciences, Şişli Hamidiye Etfal Training and Research Hospital, Department of Pathology, İstanbul, Turkey; 3 University of Health Sciences, Şişli Hamidiye Etfal Training and Research Hospital, Department of General Surgery, İstanbul, Turkey; 4 University of Health Sciences, Ankara Dışkapı Training and Research Hospital, Department of Medical Genetics, Ankara, Turkey; 5 University of Health Sciences, Şişli Hamidiye Etfal Training and Research Hospital, Department of Pediatric Radiology, İstanbul, Turkey; 6 İstanbul University İstanbul Faculty of Medicine, Department of Adolescent Medicine and Pediatrics, İstanbul, Turkey

**Keywords:** Hypoglycemia, hyperinsulinism, adolescent, multiple endocrine neoplasia 1, insulinoma, solid pseudopapillary tumor

## Abstract

Multiple endocrine neoplasia (MEN1) is a rare autosomal dominant disorder characterized by primary hyperparathyroidism, enteropancreatic neuroendocrine tumors, and anterior pituitary adenomas. A 16-year-old male presented to the emergency outpatient clinic with tonic convulsions. Physical examination in the postconvulsive period was unremarkable and revealed a muscular, postpubertal adolescent. Biochemical tests at admission were consistent with hyperinsulinemic hypoglycemia and remarkable for elevated levels of liver transaminases and creatine kinase. Work-up for a potential inborn error of metabolism and Doppler ultrasound for congenital portal-hepatic shunt were negative. When the patient was questioned, he reported using the anabolic steroid stanozolol to strengthen his muscles. His enzyme levels normalized after cessation of stanozolol. Hypoglycemia did not recur on diazoxide therapy. Magnetic resonance imaging showed two discrete lesions in the pancreas. Distal pancreatectomy revealed two masses 1.1 and 1.4 cm in diameter: a solid pseudopapillary tumor and an insulinoma. The patient also had asymptomatic primary hyperparathyroidism. DNA sequence analysis of the MEN1 gene in the index patient and his father and brother revealed a previously reported “pW183S” heterozygous mutation. This case further adds to the “pancreatic tumor” phenotype of MEN1 with the presence of a solid pseudopapillary tumor. This case report also confirms the need to meticulously question drug abuse in adolescents presenting to clinics with diagnostic challenges.

**What is already known on this topic?**

Multiple endocrine neoplasia (MEN1) is a rare autosomal dominant disorder characterized by primary hyperparathyroidism, enteropancreatic neuroendocrine tumors, and anterior pituitary adenomas. Insulinoma is a reported cause of hypoglycemia in an adolescent MEN1 patient.

**What this study adds?**

This is the first report of a case of MEN1 with a solid pseudopapillary tumor. This case report confirms the obligation to persistently question drug abuse in adolescents presenting with diagnostic challenges.

## INTRODUCTION

Multiple endocrine neoplasia (MEN1) is a rare autosomal dominant disorder characterized by primary hyperparathyroidism, enteropancreatic neuroendocrine tumors, and anterior pituitary adenomas (1). The MEN1 gene is located on chromosome 11q13 and encodes the 610-amino acid menin protein that belongs to the class of tumor suppressors (1). Because of the high degree of penetrance of the MEN1 gene, the majority of the patients develop clinical manifestations of the disorder by the fifth decade. Age-related penetrance has been studied, and the mutation seems to be non-penetrant in those younger than 5 years. It is more than 50% penetrant by 20 years of age and greater than 95% by 40 years (2). Primary hyperparathyroidism and insulinoma have been reported to occur as early as 8 and 5 years (3,4). Pancreatic involvement in asymptomatic individuals has been detected by abdominal imaging and by measurement of fasting concentrations of hormones and biomarkers such as gastrin, pancreatic polypeptide, and chromogranin A (1). Nonfunctioning pancreatic endocrine tumors have also been reported in children with MEN1 mutations (5). Herein, we describe an adolescent with MEN1 presenting with diagnostic dilemmas and the first case of a solid pseudopapillary tumor of the pancreas in a MEN1 patient.

## CASE REPORT

**Figure 1:**
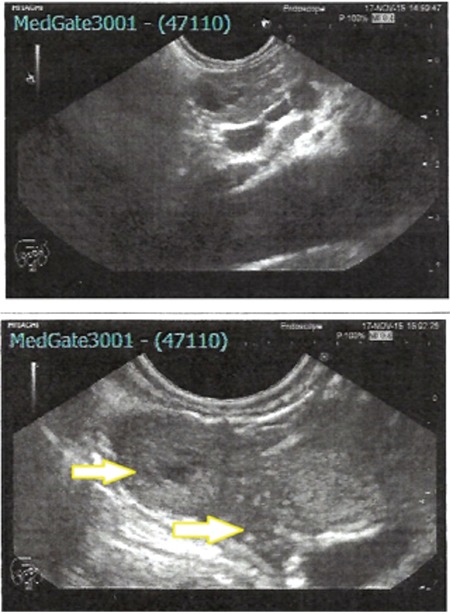
Endoscopic ultrasound images of the index patient showing the two pancreatic lesions (yellow arrows)

**Figure 2:**
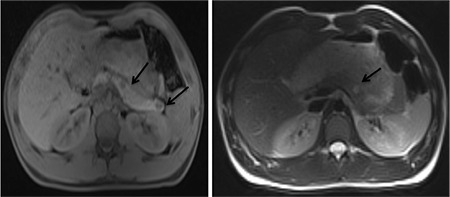
Non-contrast, T1-weighted axial pancreas magnetic resonance imaging (MRI) showing hypointense lesions (black arrows) in the tail and body of the pancreas (A). T2-weighted axial MRI revealing a hyperintense lesion (black arrow) in the body of the pancreas (B)

**Figure 3:**
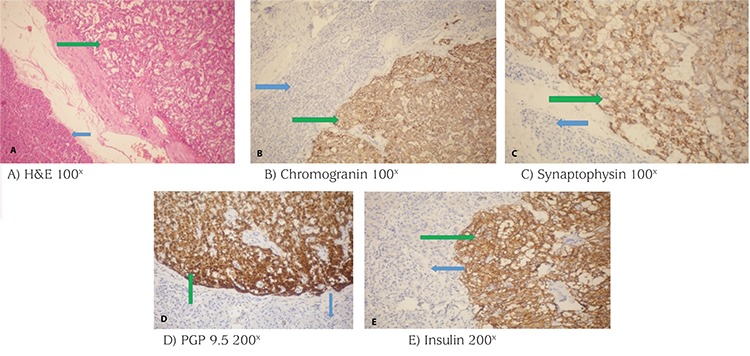
Histopathology of the patient’s distal pancreatic tumor. Hematoxylin and eosin (H&E) (A) and immunostaining (B-E) are shown. Green and blue arrows represent tumor and normal tissue, respectively. H&E revealed tumor cells with round or oval nuclei, “salt and pepper chromatin”, and an eosinophilic granular cytoplasm. The tumor nests are arranged in trabecular, insular, or sheet-like patterns. Chromogranin A, synaptophysin, protein gene product 9.5, and insulin positivity confirm the diagnosis of insulinoma H&E: hematoxylin and eosin, PGP: protein gene product

**Figure 4:**
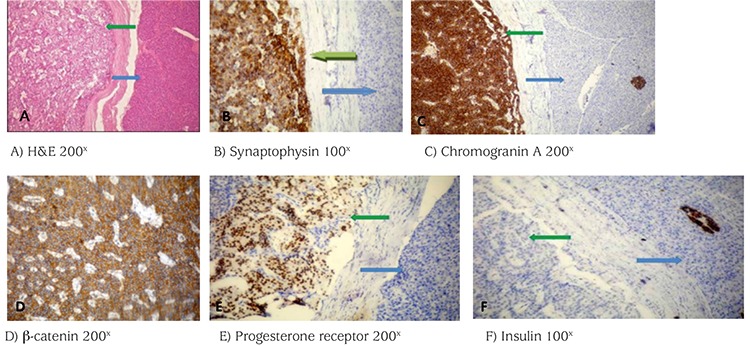
Histopathology of the patient’s tumor in the body of the pancreas. Hematoxylin and eosin (H&E) (A) and immunostaining (B-F) are shown. Green and blue arrows represent tumor and normal tissue, respectively. H&E revealed small- and medium-sized tumor cells with no atypia. Pseudopapillary structures were observed in most areas. The tumor stained positive for synaptophysin (B), chromogranin A (C), β-catenin (D), and progesterone receptor (E) but was negative for insulin (F); these findings confirm a solid pseudopapillary tumor of the pancreas H&E: hematoxylin and eosin

A 16-year-old male adolescent with a diagnosis of primary epilepsy was referred to our center due to tonic convulsions. Family history was significant for maternal death owing to metastatic lung adenocarcinoma and a paternal history of low-grade liposarcomas and recurrent nephrolithiasis. Physical examination was unremarkable and revealed a well-built, muscular adolescent. Biochemical tests at admission were consistent with hyperinsulinemic hypoglycemia as follows: venous blood glucose, 24 mg/dL; cortisol, 18 µg/dL; growth hormone, 13 ng/mL; insulin, 8 µU/mL; and C-peptide, 1.34 ng/mL. Anti-insulin antibodies were negative. Serum liver transaminase and creatine kinase levels were elevated. Alanine amino transferase was 349 U/L; aspartate aminotransferase, 158 U/L; and creatine kinase 834 U/L. The metabolic work-up for inborn errors of metabolism was negative; tandem mass spectrometry findings and urinary amino acid and organic acid levels were normal. Hepatic ultrasonography for a congenital portal-hepatic shunt was negative. Thin-slice pancreas computed tomography and pancreas magnetic resonance imaging (MRI) findings were normal. On further questioning, the patient admitted taking stanazolol (Winstrol) to strengthen his muscles. Liver transaminase and creatine kinase levels normalized within 3 weeks of stanozolol cessation. Hypoglycemia did not recur on diazoxide (200 mg/day) therapy. Endoscopic ultrasound to search for an insulinoma was scheduled, but the patient refused to undergo endoscopic ultrasound for 7 months. Biochemical evaluation also revealed asymptomatic primary hyperparathyroidism. Serum calcium level was 11.5 mg/dL; phosphorus, 2.6 mg/dL; alkaline phosphatase, 520 IU/L; parathyroid hormone, 320 pg/mL; 25-hydroxyvitamin D, 33 ng/mL; and 24-h urinary calcium, 4.2 mg/kg/day. Parathyroid scintigraphy revealed an adenoma in the inferior right parathyroid gland. The constellation of primary hyperparathyroidism and hyperinsulinemic hypoglycemia due to a possible insulinoma prompted us to search for a mutation in the menin gene. DNA sequence analysis of the menin gene revealed a previously reported heterozygous “pW183S” mutation at codon 183 on exon 3 of the menin gene located at chromosome 11 q13 (6). Plasma fasting gastrin and glucagon levels were within normal ranges. Screening of the father and the 14-year-old brother of the proband revealed the same mutation. The brother had asymptomatic hyperparathyroidism. Endoscopic ultrasound of the patient revealed two masses: one in the body (1.4 cm) and the other in the tail of the pancreas (1.1 cm) (Figure 1). Repeat pancreas MRI prior to surgery confirmed the presence of these masses (Figure 2). Histopathologic evaluation of the mass in the tail of the pancreas revealed tumor cells with round/oval nuclei and eosinophilic granular cytoplasm. The tumor nests were arranged in trabecular, insular, or sheet-like patterns (Figure 3A). Immunocytochemical evaluation of the mass in the tail of the pancreas was consistent with a grade 1 neuroendocrine tumor according to World Health Organization classification and stained positive for chromogranin, synaptophysin, protein gene product 9.5, and insulin (Figure 3B, 3C, 3D, 3E). Ki-67 index was less than 1%. Thus, the distal mass was diagnosed as an insulinoma. The proximal region mass was composed of small- and medium-sized tumor cells with no apparent atypia. Pseudopapillary structures were observed in most of the areas (Figure 4A). The tumor was positive for β-catenin, CD56, progesterone receptor, chromogranin, and synaptophysin (Figure 4). The proximal mass was diagnosed as a solid pseudopapillary tumor. Ki-67 index was 6% to 7%. Staining of both tumors was negative for menin (images are unavailable since they were processed at another center which did not share the slides).

Postoperatively, hypoglycemia did not recur when diazoxide was discontinued. Conversely, the patient developed diabetes and normoglycemia was restored with insulin glargine at a mean dose of 0.45 U/kg/day. His requirement for insulin diminished 8 months postoperatively. Informed consent was taken from the father of the patient, and assent was also taken from the patient.

## DISCUSSION

The present case has unique aspects regarding the biochemical evaluation for hypoglycemia and the puzzling histopathologic findings of a solid pseudopapillary tumor and an insulinoma following distal pancreatectomy and splenectomy.

The etiology of hypoglycemia in the adolescent includes a variety of disorders reviewed elsewhere (7). Insulinoma is not an infrequent cause of hypoglycemia in adolescents and its presence should always prompt consideration of MEN1. The use of stanazolol in the current case further complicated the evaluation owing to elevated liver transaminase and creatine kinase levels, which prompted us to search for inborn errors of metabolism and congenital portosystemic shunts (Abernethy malformation), which have recently been reported as a cause of hyperinsulinemic hypoglycemia (8). In the current case, repeated interviews uncovered drug abuse as the cause of the discordant biochemical anomalies. Adolescence is a particularly vulnerable period for drug abuse that should always be considered as a reason for unusual clinical and/or biochemical findings that present diagnostic challenges.

To the best of our knowledge, this is the first report of a solid pseudopapillary tumor of the pancreas in a MEN1 patient. Moreover, this is also the first description of a synchronous nonfunctional tumor and insulinoma in a pediatric MEN1 patient. In the literature, there are two adult case reports of solid pseudopapillary tumor of the pancreas and functioning adenomas of the pancreas (9,10). However, these cases were not clinically or genetically diagnosed as having MEN1. Solid pseudopapillary tumor of the pancreas is very rare constituting less than 1% of all pancreatic masses (11). The cellular origin of the tumor is uncertain. Some theories suggest that these tumors originate from small duct epithelium, acinar or pluripotent stem cells capable of exocrine and endocrine differentiation (11). They are of benign nature or have a low-grade malignancy potential that typically occurs in young women. These tumors are very unusual in males. The tumor can be located throughout the pancreas. Although it is generally benign or has low-grade malignant potential, it may invade surrounding soft tissues and can even metastasize to the liver or peritoneum in rare cases. The tumor is a combination of solid growth pattern and cystic areas of loosely cohesive epithelioid cells that can become hemorrhagic. Microscopically, the tumors are positive for β-catenin, CD56, synaptophysin, and progesterone receptors (12). Nonfunctional pancreatic tumors have been associated with increased mortality in MEN1 patients.

Genetically confirming a clinical diagnosis of MEN1 in the proband is mandatory to assess other family members for the presence of the mutation so that they can be followed for potential endocrine problems and for avoiding unnecessary work-up due to “phenocopy.” In the present case, the 14-year-old brother of the proband had asymptomatic hyperparathyroidism. The presence of the mutation indicates that he also needs lifetime surveillance for occurrence of MEN1-related tumors.

Drug abuse should be considered in adolescents presenting to clinics, particularly when the diagnosis is unclear. This case report adds to the “pancreatic tumor” phenotype of MEN1 with a solid pseudopapillary tumor.
